# Improving the Neighborhood Environment for Urban Older Adults: Social Context and Self-Rated Health

**DOI:** 10.3390/ijerph13010003

**Published:** 2015-12-22

**Authors:** Arlesia Mathis, Ronica Rooks, Daniel Kruger

**Affiliations:** 1 Institute of Public Health, Florida A&M University, 1515 MLK Boulevard, Tallahassee, FL 32307, USA; 2 Department of Health and Behavioral Sciences, University of Colorado Denver, P.O. Box 173364, CB 188, Denver, CO 80217, USA; ronica.rooks@ucdenver.edu; 3 Prevention Research Center of Michigan, University of Michigan School of Public Health, 1420 Washington Heights, Ann Arbor, MI 48109, USA; kruger@umich.edu

**Keywords:** neighborhoods, older adults, urban health, social capital, crime

## Abstract

*Objective*: By 2030, older adults will account for 20% of the U.S. population. Over 80% of older adults live in urban areas. This study examines associations between neighborhood environment and self-rated health (SRH) among urban older adults. *Methods*: We selected 217 individuals aged 65+ living in a deindustrialized Midwestern city who answered questions on the 2009 Speak to Your Health survey. The relationship between neighborhood environment and self-rated health (SRH) was analyzed using regression and GIS models. Neighborhood variables included social support and participation, perceived racism and crime. Additional models included actual crime indices to compare differences between perceived and actual crime. *Results*: Seniors who have poor SRH are 21% more likely to report fear of crime than seniors with excellent SRH (*p* = 0.01). Additional analyses revealed Black seniors are 7% less likely to participate in social activities (*p* = 0.005) and 4% more likely to report experiencing racism (*p* < 0.001). *Discussion*: Given the increasing numbers of older adults living in urban neighborhoods, studies such as this one are important for well-being among seniors. Mitigating environmental influences in the neighborhood which are associated with poor SRH may allow urban older adults to maintain health and reduce disability.

## 1. Introduction

During the last decade, there has been a resurgence of interest in the impact of neighborhoods on health. Growing epidemiological and sociological evidence link the residential environment to an individual’s health [1,2]. The effect of neighborhood on health is particularly salient among older adults because older individuals are most likely the longest dwelling residents in the community and they have increased reliance on resources in their immediate neighborhoods [3]. Poor neighborhood conditions, which include a lack of social support, social networks, social cohesion and low perceptions of safety [4], may contribute to physical inactivity [5], obesity [6], and mental health disorders [1]. 

The geographic area mostly commonly referred to as the neighborhood is not precise. In health research, the terms neighborhood and community are often used interchangeably to refer to a person’s immediate residential environment which is hypothesized to have material and social characteristics related to health [3]. Administratively defined areas, such as census tracts, block groups, and zip (postal) codes have been used as rough proxies for neighborhoods. Other criteria used to define a neighborhood can be historical, based on residential characteristics, or based on people’s perceptions [7]. The size and definition of the relevant geographic area may vary according to the outcomes being studied. For example, neighborhoods defined on the basis of people’s perceptions may be relevant when the characteristics of interest relate to social interaction or social cohesion [8]; however administratively defined neighborhoods may be relevant when researching policies [9]; and geographically defined neighborhoods may be relevant when features of the physical environment are theorized to be important [10].

This study uses self-rated health (SRH) to evaluate the state of health among older adults living in urban neighborhoods. Included in research studies since the 1950s, the concept of SRH is useful in documenting the current state of health self-reported by older adults and also in predicting future health-related events [11]. SRH integrates information on the biological, mental, functional, and spiritual dimensions of the person’s health [11]. Our study, in addition to others, suggests that SRH may be influenced by demographics [12] and neighborhood factors [8]. In addition to reflecting overall health status in older adults, SRH can provide information to assist in the development and implementation of community health promotion and disease prevention programs as well as planning and providing adequate levels of care for older adults.

The purpose of this study is to examine associations between the neighborhood environment and SRH in older adults living in an urban environment. This study is based on a theoretical framework for understanding social inequalities in health and aging proposed by House [13]. House graphically depicts how social, political, and economic conditions and policies as well as ascribed status and position in terms of race, gender, and age are believed to affect a wide range of health outcomes (including SRH) among older adults. SRH is a sensitive measure of overall health in older adults [11]. Similar to Diez-Roux’s neighborhood model [3] which suggests that disadvantaged neighborhoods may lead to poor health outcomes, House proposes that social and environmental hazards such as lack of safety particularly at home may “get under the skin” causing changes in blood pressure or immune response. Our study uses variables from House’s framework to examine how neighborhood environment, which includes social support/participation, fear of crime, and perceived racism affect SRH among older adults. Our study also examines how demographic characteristics such as race and gender affect older adults’ perceptions (fear) of crime in comparison to actual neighborhood crime. 

## 2. Methods

This study uses secondary data extracted from the 2009 Speak to Your Health Community survey [14]. Speak to Your Health is a telephone survey conducted by the Prevention Research Center of Michigan to collect demographic, environmental (*i.e.*, neighborhood characteristics), services, and health information from a cross-section of individuals living in Genesee County, Michigan (USA). The survey uses random digit dialing to select a sample of households throughout the county. The Prevention Research Center of Michigan is a community-university partnership which includes the University of Michigan, School of Public Health, the Genesee County Health Department and the Greater Flint Health Coalition. Additional details on the Speak to Your Health survey were published in an earlier article [15]. 

The study also used crime statistics from Location, Inc. (Worcester, MA, USA) which is a provider of location-based statistical data that includes crime statistics, lifestyle and demographic data on neighborhoods across the United States. The actual crime indices for each neighborhood are based on data from the Federal Bureau of Investigations (FBI) and the U.S. Justice Department. The crime indices used in this study are the same as the FBI defined crime index which is composed of the eight offenses the FBI combines to produce its annual index. These offenses include willful homicide, forcible rape, robbery, burglary, aggravated assault, larceny, motor vehicle theft, and arson. 

### 2.1. Setting

Flint, Michigan, the urban center of Genesee County, is a de-industrialized city whose economy and population declined during the latter part of the twentieth century. Flint has high unemployment and based on local crime rates, was recently ranked in the top five most dangerous cities in the United States [15].

### 2.2. Subjects

From 1698 participants who answered questions on the 2009 Speak to Your Health survey, we focused on the 217 individuals over 65 years of age who lived within the city of Flint. Because of the low number of survey participants from other races (<5), we selected only White and Black participants and stratified the study subjects by racial categories. The terms Black and African American are used interchangeably and refer to the same group. Basic demographic characteristics collected as background information on participants included age, gender, education, marital status, and health status (see Table 1).

The proposal for this study was submitted for review to the university’s IRB and was determined to be exempt because of its use of de-identified survey data. The survey committee of the Prevention Research Center of Michigan also reviewed the manuscript to evaluate appropriate use of the data.

**Table 1:** Descriptive statistics of the study population.

Total (N = 217)	White	African American	Range
**Demographics**			
Race (N)	113	104	
Age **^a^** (years)	74.26	74.18	65–91
Gender (%)			
Male	30	27	
Female	70	73	
**Social/Economic**			
Education (%)			1–4
< High school	7	27	
High School or GED	40	29	
College/Tech/Assoc. Degree	30	28	
Bachelor’s or Above	22	15	
Marital Status (%)			0–1
Single	67	59	
Married/committed relationship	32	40	
**Health Status**			
Self-rated Health **^b^**			1–3
Excellent	28.8	31.7	
Average	31.5	32.7	
Poor	39.6	35.6	
No. of Chronic Conditions **^c^**	2.22	2.08	0–5
Psychological Conditions **^d^**	<1.0	<1.0	0–3
**Neighborhood**			
Social Support	7.73	7.13	6–12
Social Participation	8.96	8.32	5–10
Racism	6.66	8.86	5–17
Perceived Crime	8.95	8.83	4–17
Actual Crime Rate **^e^**			
High	46.0	62.7	1–40
Moderate	31.9	28.8	41–60
Low	22.1	8.7	61–100

Note: **^a^** The average age in years is reported for each group; **^b^** The self-rated response percentage is reported for each category by group; **^c^** The average number of chronic conditions is reported for each group; **^d^** The average number of psychological conditions is reported for each group; **^e^** The percentage of each response is reported for each category by group.

### 2.3. Outcome Measure

#### Self-Rated Health

Individuals were assessed on health status using self-report indicators (See Table 1). The Speak to Your Health survey asked subjects to self-rate their health. The indicators *excellent*, *very good*, *good*, *fair*, and *poor* were then converted to numeric values 1 through 5 with higher values indicating excellent health. For our analysis, we combined subjects who rated their health as excellent or very good into a single category labelled excellent self-rated health (SRH). We also merged subjects who rated their health as fair or poor into one category labelled poor self-rated health (SRH).

### 2.4. Neighborhood Measures

#### 2.4.1. Social Capital

To measure individual and collective social capital, the researchers selected eleven items from the Speak to Your Health survey. Social support (individual social capital) was measured by using six items from the Speak to Your Health survey. Individuals were asked about their relationships with relatives, friends, community members, and the religious community [16]. Social participation (collective social capital) was measured with five items. Survey respondents were asked if they were “involved in neighborhood clean-up, beautification, or community garden project,” “involved in meeting of a block or neighborhood group,” “took action with neighbors to do something about a neighborhood problem,” and “volunteer in a program at a local school” [16]. 

#### 2.4.2. Actual Neighborhood Crime

The crime rate in the neighborhood was measured using an index which ranged from 1 to 100 with 1 being the most dangerous. Crime indices for each neighborhood were based on data from the Federal Bureau of Investigations (FBI) and the U.S. Justice Department. The crime indices used in this study gathered from Location, Inc. are the same as the FBI defined crime index composed of eight offenses the FBI combines to produce its annual index. 

#### 2.4.3. Perception of Neighborhood Crime

Perceptions of neighborhood crime and safety were assessed with an item collected from the survey. Survey responses were collected from the following question: ‘‘How fearful are you about crime in your neighborhood?’’*(very fearful*, *somewhat fearful*, *not very fearful*, *and not at all fearful)*; ‘‘How safe is it to walk around alone in your neighborhood during the daytime?’’ *(extremely dangerous, somewhat dangerous, fairly safe, completely safe)*; and ‘‘How safe is it to walk around alone in your neighborhood after dark?’’ *(extremely dangerous*, *somewhat dangerous*, *fairly safe*, *completely safe)*. The response indicators were then converted to numeric values 1 through 4 with high values indicating very fearful or extremely dangerous. For the final item, ‘‘Compared to other neighborhoods, the crime rate in my neighborhood is” *(very high, high, about the same, low, and very low)*, the indicators were converted to numeric values 1 through 5 with higher values indicating very high or high crime. While using crime rates in a neighborhood is a more objective measure of neighborhood safety, subjective experiences and perceptions are more directly related to health [17] and are highly correlated with objective measures [4,18,19,20].

#### 2.4.4. Perceived Racism

We assessed perceived racism using multiple items [21]. Respondents indicated the degree to which they were ignored, overlooked, or not given services, were treated rudely or disrespectfully, and were treated as if they were “stupid” or “talked down to” because of their race *(never*, *rarely*, *sometimes*, *or often)*.

### 2.5. Health Status

The study included two additional physical and mental health outcome measures because of their relationship to SRH. Subjects were also asked whether or not they had been diagnosed with high blood pressure, heart disease, stroke, cancer, and diabetes (yes or no). For our analysis, each subject received a score based on the number of chronic conditions reported. For the final assessment of health status, we measured our study population on psychological conditions. Subjects reported (yes or no) whether they had been diagnosed with depression, anxiety or sleep disorders. As with our assessment of chronic conditions, each subject received a score based on the number of psychological conditions reported.

### 2.6. Demographic Variables

Demographic variables collected for our study population included race, gender, age, education, and marital status. Race included only Black and White participants because of the limited number of other races (<5). Age was examined as both a continuous and a categorical variable. The researchers subdivided the older adults into three groups—younger old (ages 65–74), older old (ages 75–84) and oldest old (ages 85+). In addition to looking at variations among populations by gender and race, researchers also examined differences between older adults from youngest old to oldest old. Education was collected as a categorical variable. For the purpose of this study, it was collapsed into four categories: less than high school, high school graduate, some college/technical school/associate’s degree, and bachelors’ degree or above. The study also included marital status—single (includes divorced, or widowed) and married or in a committed relationship.

### 2.7. Statistical Analyses

**Figure 1:**
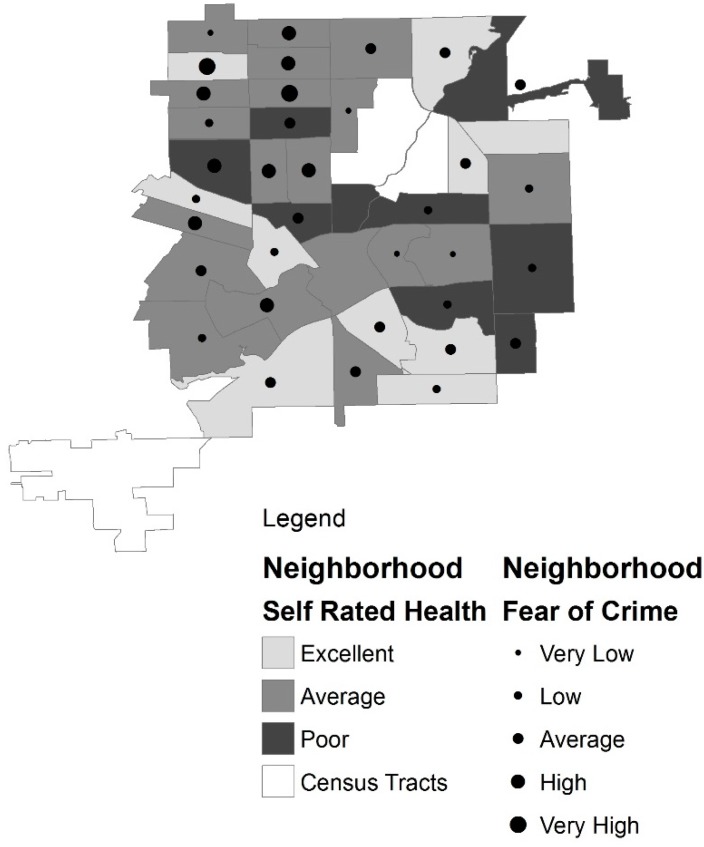
Flint neighborhoods by self rated health and fear of crime.

We stratified our study population by race (White and African American) and calculated the average age for each group. Then we assessed the proportions of each group based on gender, education level and marital status (see Table 1). For the analysis of the neighborhood environment on SRH, we used a multinomial logistic regression model which included SRH as our variable of interest with demographic, socio-economic, neighborhood and health status variables. We used psychological and chronic conditions as health status variables in the model to control for differences between groups in addition to controlling for their effect on self-rated health (see Table 2). Because results of our analysis showed a strong association between poor SRH and fear of crime, we conducted additional analyses to examine this relationship. We used ArcGIS software to map the relationship between SRH and fear of crime by neighborhood (see Figure 1). Neighborhoods are shown at the census tract level. The neighborhood representation for fear of crime takes the average of all older survey participants living within the neighborhood. The average score of each neighborhood was assigned a category of very low, low, average, high, or very high. The neighborhood then received a symbol which was sized according to the category. Larger dots symbolized greater fear of crime. An additional GIS layer shows SRH. SRH takes the average of all older survey participants living within the neighborhood. The neighborhood then receives a color based on the average SRH. Darker colors represent poorer health. For further statistical analyses of fear of crime in our study population, we also used a Poisson regression model (see Table 3). The model included fear of crime as our variable of interest. Similar to our previous model, we included demographic, socio-economic, neighborhood and health status variables. Because this analysis revealed differences based on race, we stratified the subjects by race and re-analyzed the data for each racial group. We reported adjusted odds ratios, confidence intervals, and p-values for each group.

**Table 2:** Neighborhood environment and self-rated health.

Variables	Excellent SRH *vs.* Poor SRH			Excellent SRH *vs.* Average SRH		
	OR	95% CI	*p*-Value	OR	95% CI	*p*-Value
*Demographic*						
Race						
White	1.00	Reference		1.00	Reference	
Black	0.85	0.34–2.16	0.74	0.63	0.27–1.48	0.29
Gender						
Male	1.00	Reference		1.00	Reference	
Female	0.57	0.21–1.51	0.26	0.66	0.28–1.57	0.35
*Social/Economic*						
Marital Status						
Single *	1.11	0.47–2.66	0.81	1.30	0.59–2.85	0.51
Married	1.00	Reference		1.00	Reference	
Education						
< High School	5.12	1.10–23.89	0.03	2.85	0.82–9.91	0.09
High School Graduate	4.60	1.33–15.86	0.02	1.46	0.54–3.97	0.46
College/Associate Deg.	2.16	0.59–8.01	0.25	1.05	0.39–2.83	0.92
Bachelor’s and Above	1.00	Reference		1.00	Reference	
*Neighborhood Environment*						
Social Support	0.87	0.65–1.17	0.35	0.80	0.60–1.06	0.12
Social Participation	1.33	0.96–1.85	0.08	1.01	0.77–1.34	0.90
Racism(Chronic Stress)	0.66	0.84–1.12	0.97	1.06	0.93–1.20	0.36
Fear of Crime	1.21	1.05–1.39	0.01	1.10	0.96–1.25	0.14
*Health Outcome*						
Psychological Conditions	3.18	1.65–6.12	0.001	1.69	0.87–3.26	0.12
Chronic Conditions	2.88	1.84–4.49	<0.001	1.34	0.90–2.00	0.15
N	213					

Notes: * Single includes widowed, separated and divorced participants.

For our final analysis, we wanted to evaluate whether actual crime in the community had the same relationship to SRH as fear of crime (see Table 4). To analyze this data, we use a multinomial logistic regression model which included all variables in our initial analysis, except we replaced fear of crime with actual crime rate categories (low, medium, and high). All regression models were analyzed using SPSS, version 19 (IBM, Armonk, NY, USA).

## 3. Results

Our study population consisted of 217 individuals ranging in age from 65 to 91 years of age. The average age was similar for Whites and Blacks at 74.26 and 74.18 years respectively. Among White seniors, 70% were female and 73% among Blacks. Seven percent did not complete high school or GED compared with 27% of Blacks. Of those with the highest levels of education, 22% of Whites held a bachelor’s degree or higher, while 15% of Blacks had an equivalent level of education. Only 32% of Whites were married or in a committed relationship compared with 40% of Blacks. There were no significant differences between White and Black seniors on the previous demographic variables. The mean scores for SRH were not significantly different between Whites (2.10) and Blacks (2.04). For psychological conditions, 37.5% of White seniors reported one or more of the listed conditions, but only 24% of Black seniors did the same. Finally, the average number of chronic conditions was not significantly different (2.22 for Whites and 2.08 for Blacks). 

Figure 1 shows the relationship between neighborhood SRH and fear of crime. For the GIS analysis, we combined scores of individuals living within each census tract and then divided by the number of participants within that neighborhood to get the average (mean) SRH for the neighborhood. This created a standardized unit for each neighborhood. We summarized SRH into three categories. The neighborhoods with excellent SRH had scores between 3.6 and 4.4. The scores for average SRH were between 2.7 to 3.5. The neighborhoods with scores from 1.5 to 2.6 were labelled as poor SRH. For the fear of crime data we also combined the scores of the individuals living within each of the census tracts and divided by the number of participants within that tract to get a score for the tract. Categories were created to summarize the data. The very low category scores fell between 5.8 and 7.0. The low scores were between 7.0 and 8.6. The average scores were 8.7 to 9.7. The high scores were 9.8 to 11.0. The very high scores were 11.1 to 12.0. There were no average neighborhood scores lower than 5.8 or above 12.0. We used Jenks natural breaks to determine the cut points for both SRH and fear of crime categories. 

Table 2 summarizes our analysis of neighborhood environment and self-rated health among older adults. Seniors with poor SRH were 21% more likely to report fear of crime compared with seniors with excellent SRH (*p* = 0.01). They were also twice as likely to have chronic conditions and three times more likely to report psychological conditions such as depression, anxiety, or sleep disorders. Seniors with poor SRH were also 4 to 5 times more likely to have a high school education or less. Because of the significant relationship between fear of crime and poor SRH, we conducted further analysis of this effect. Results are shown in Table 3. 

**Table 3:** Fear of crime and race.

Variables	Fear of Crime Total			White			Black		
	OR	95% CI	*p*-value	OR	95% CI	*p*-Value	OR	95% CI	*p*-Value
*Demographic*									
Race									
White	1.00	Reference							
Black	0.87	0.78–0.97	0.01						
Gender									
Male	1.00	Reference		1.00	Reference		1.00	Reference	
Female	1.03	0.93–1.14	0.58	1.02	0.90–1.18	0.73	1.05	0.89–1.23	0.30
*Social/Economic*									
Marital Status									
Single	1.01	0.92–1.12	0.78	1.09	0.94–1.27	0.25	0.91	0.79–1.05	0.22
Married	1.00	Reference		1.00	Reference		1.00	Reference	
Education									
< High School	1.18	1.00–1.38	0.04	0.99	0.75–1.30	0.95	1.38	1.09–1.75	0.01
High School Graduate	1.13	0.98–1.29	0.08	1.10	0.93–1.30	0.26	1.17	0.94–1.46	0.33
College/Assoc. Degree	1.12	0.97–1.28	0.13	1.06	0.88–1.27	0.55	1.21	0.69–1.50	0.18
Bachelor’s and Above	1.00	Reference		1.00	Reference		1.00	Reference	
*Neighborhood Environment*									
Social Support	1.02	0.98–1.05	0.28	1.01	0.97–1.06	0.59	1.03	0.98–1.08	0.24
Social Participation	0.94	0.92–0.98	<0.005	0.95	0.90–1.00	0.06	0.93	0.89–0.98	0.005
Racism (Chronic Stress)	1.03	1.02–1.05	<0.001	1.03	1.00–1.06	0.04	1.04	1.01–1.05	<0.001
*Health Outcome*									
Psychological Conditions	1.03	0.97–1.09	0.32	1.04	0.96–1.13	0.30	1.00	0.92–1.09	0.93
N	213			110			103		

**Table 4:** Self-rated health and actual neighborhood crime.

Variables	Excellent SRH *vs.* Poor SRH			Excellent SRH *vs.* Average SRH		
	OR	95% CI	*p*-Value	OR	95% CI	*p*-Value
*Demographic*						
Race						
White	1.00	Reference		1.00	Reference	
Black	0.64	0.25–1.63	0.35	0.56	0.24–1.31	0.18
Gender						
Male	1.00	Reference		1.00	Reference	
Female	0.64	0.25–1.65	0.35	0.74	0.31 –1.67	0.44
*Social/Economic*						
Marital Status						
Single *	1.06	0.45–2.50	0.84	1.29	0.59–2.83	0.52
Married	1.00	Reference		1.00	Reference	
Education						
< High School	6.39	1.38–29.64	0.02	3.32	0.93–11.81	0.06
High School Graduate	5.29	1.54–18.18	<0.01	1.62	0.61–4.44	0.33
College/Associate Deg.	2.42	0.66–8.94	0.18	1.13	0.41–3.13	0.80
Bachelor’s and Above	1.00	Reference		1.00	Reference	
*Neighborhood Environment*						
Social Support	0.90	0.66–1.19	0.44	0.80	0.60–1.06	0.11
Social Participation	1.19	0.87–1.62	0.28	0.96	0.73–1.26	0.96
Racism (Chronic Stress)	1.03	0.89–1.18	0.68	1.09	0.96–1.22	0.18
Actual Crime Rate						
High	0.73	0.22–2.43	0.61	0.87	0.30–2.50	0.80
Moderate	1.19	0.47–3.05	0.71	1.24	0.41–3.74	0.70
Low	1.00	Reference		1.00	Reference	
*Health Outcome*						
Psychological Conditions	3.18	1.69–5.97	<0.001	1.65	0.87–3.14	0.13
Chronic Conditions	2.95	1.89–4.61	<0.001	1.37	0.92–2.04	0.13
N	213					

Notes: * Single also includes widowed, separated and divorced participants.

Table 3 shows fear of crime as our outcome measure. For seniors overall, social participation (*p* < 0.005) and racism (*p* < 0.001) are strongly associated with fear of crime. Seniors who report fear of crime are 6% less likely to participate in social activities in the neighborhood and 3% more likely to experience racism. Also, these seniors are 18% more likely to have lower levels of education (less than high school). Results also show there is also a racial difference between Black and White seniors (*p* = 0.01) reporting fear of crime. Black seniors are 7% less likely to engage in social activities in their neighborhood (*p* = 0.005) and 4% more likely to report racism (*p* < 0.001). Although White seniors are less likely to participate in neighborhood activities, the difference is not significant. However, like Black seniors, they are more likely to report racism (*p* = 0.04). Furthermore, Black seniors reporting fear of crime are 38% more likely to have less than a high school education.

Our final model examined associations between actual neighborhood crime and SRH. Again, we used SRH as our outcome measure. When we added actual neighborhood crime indices to our model, we found no significant relationship between actual crime and SRH. However, as expected, we found a relationship between SRH, chronic and psychological conditions and level of education. Seniors with poor SRH health were twice as likely to have chronic conditions and 3 times more likely to report psychological conditions. In addition, seniors with poor SRH were 5 to 6 times more likely to have a high school education or less.

## 4. Discussion

In summary, fear of crime was strongly related to poor SRH among older adults. This study supports the conceptual framework for understanding social inequalities in health and aging proposed by House [13]. This framework, based on a stress and adaptation model from social epidemiology, theorizes that socioeconomic position and race/ethnicity shape individuals exposure to and experience of virtually all known psychosocial and environmental risk factors. These risk factors explain the size and persistence of social disparities in health. Our study also supports similar findings between neighborhood factors and psychological distress [19]. Booth *et al*. found that neighborhood factors are associated with mental health outcomes, but concluded that more research was needed. Both studies support the social stress theory that chronic stressors outside the individual become internalized. Our findings provide additional information to previous studies by showing a relationship between fear of crime and poor SRH among older adults. 

Our study built on these previous studies by also comparing fear of crime and actual crime indices to poor SRH among seniors. Although actual rates of crime victimization among older adults is much lower than younger people [18], older individuals express higher levels of fear of crime and lower levels of perceived safety [19]. Among both White and Black seniors, this results in lower social participation and perceptions of higher levels of racism. The relationship was even stronger among Black seniors. Furthermore, Black seniors with less than a high school education are 38% more likely (*p* = 0.01) to fear crime in their community. This study also shows although women are more likely than men to experience fear of crime, the differences were not significant. We are unable to say from these results whether increased fear causes lack of participation or whether increased fear is the result of lack of involvement in neighborhood activities. Individuals who are involved in neighborhood activities are more likely to meet their neighbors and establish relationships. Previous studies have shown that knowing one’s neighbors can decrease vulnerability to health risk by increasing social capital. The strength of this study is that it uses subjective and objective measures of the neighborhood’s environment, particularly for crime. However, several limitations of the study should be noted. 

The first limitation is that the data are cross-sectional. Because of the cross-sectional data, we cannot infer that the findings of the study are causal in nature. However, the evidence is consistent with the conceptual frameworks of Diez-Roux [3], which suggests that a disadvantaged neighborhood environment is related to poor health, and House [13], which suggests that neighborhood environment can and does “get under the skin” causing biological changes which increase poor health. More studies are needed to further analyze the reciprocal influences of neighborhood as a place of residence, perceptions of the neighborhood, and self-rated health. The second limitation of the study is that although our sample is population based, our analysis is focused on only Black and White urban residents over the age of 65 living in Flint, Michigan. The study could be strengthened by examining additional races or ethnicities in urban centers in other regions. However, because of the characteristics of this geographic area, the numbers of participants of other races or ethnicities was too small to analyze. But, metropolitan areas, especially in the Midwest, are similar in population characteristics and socioeconomic structure. Also, the contextual neighborhood effects have been examined in other settings [8,22,23]. Therefore, we believe that this study may provide information for other urban settings with inequities in neighborhood environments. In addition, self-rated health rather than actual measures of health as an outcome variable may have introduced response bias. People in poor health may feel more negative about their neighborhoods [8]. Although these findings give insight into the relationship between fear of crime and SRH, it still raises questions that should be explored—mainly is there a causal link between fear of crime and poor SRH. Future research should assess what creates fear of crime and whether this fear substantially changes an individual’s health behaviors or health status. It is reasonable to assume that fear is caused by objective measures such as actual neighborhood crime, but it appears that other influences such as the media, neighborhood history, and/or history of victimization may play greater role. 

### Implications for Neighborhood Environment and Health 

Aging populations within urban neighborhoods will create a series of challenges to the provision of health and social care. As the population ages, the total amount of ill health and disability in the population will increase unless there is considerable improvement in the health of current and future urban seniors [24]. These changes are expected to occur because of the shift from acute infectious disease to complex chronic long-term illness and disability. This shift is expected to cause dramatic changes in the allocation of health care resources and the configuration of services [25]. It has also been predicted that even if increases in the urban older adult population does not exert pressure for additional resources in the health care system, it may create the need for the development and improvement of community services for seniors with complex health needs. Mitigating environmental influences in the neighborhood which are associated with poor SRH may allow urban older adults to maintain health and reduce disability. Extending healthy lives within this population will reduce costs associated with long-term health and social care [24]. 

## 5. Conclusions

A growing body of literature has reported associations between neighborhoods and health [26]. As the literature expands, it is worthwhile to consider populations such as older adults because the proportion of people aged 65 and older is growing. In the U.S., the number of people over the age of 65 is expected to reach 72 million—which will account for roughly 20% of the population [6]. Previous research relating neighborhoods to health in older adults examined mortality [9], mental health [27], and health behaviors [28]. This study adds to existing literature by examining perceived *vs.* actual effects of neighborhood environment among urban older adults. We were able to show a relationship between fear of crime and poor SRH among urban seniors. In addition, we were able to show that poor SRH is not related to objective measures of actual crime but is related to perception of crime. This suggests that self-rated health is more affected by perception of the neighborhood. This finding supports the suggestion that self-rated health may be improved by improving senior adults’ attitudes about their neighborhood environment. Specific strategies may include reducing fear by creating activities which focus on meeting other individuals in the neighborhood. We also found that race is a determinant in older peoples’ perceptions. Understanding specific neighborhood influences on health will enable us to improve the lives of older adults, many of who are aging in place [29], and is crucial in addressing growing populations of urban older adults. 
